# Phylogenomic Insights into Diversity and Evolution of Nonpathogenic *Xanthomonas* Strains Associated with Citrus

**DOI:** 10.1128/mSphere.00087-20

**Published:** 2020-04-15

**Authors:** Kanika Bansal, Sanjeet Kumar, Prabhu B. Patil

**Affiliations:** a Bacterial Genomics and Evolution Laboratory, CSIR-Institute of Microbial Technology, Chandigarh, India; National Institute of Advanced Industrial Science and Technology

**Keywords:** nonpathogenic *Xanthomonas*, evolution, ecology, pathogenicity, comparative genomics, virulence- and plant adaptation-related gene clusters, speciation

## Abstract

*Xanthomonas* species are primarily known as a group of phytopathogens infecting diverse plants. Recent molecular studies reveal the existence of potential novel species and strains of *Xanthomonas* following a nonpathogenic lifestyle. In the present study, we report whole-genome sequences of four nonpathogenic strains from citrus (NPXc). Taxonogenomics revealed the surprising diversity, as each of these three isolates were found to be potential novel species that together form a citrus-associated nonpathogenic *Xanthomonas* species complex (NPXc complex). Interestingly, this NPXc complex is related to another nonpathogenic species, Xanthomonas sontii, from rice (NPXr). On the other hand, the fourth NPXc isolate was found to be related to nonpathogenic isolates from walnut (NPXw); altogether, they form a potential taxonomic outlier of pathogenic Xanthomonas arboricola species. Furthermore, genomic investigation of well-characterized pathogenicity clusters in NPXc isolates revealed lifestyle-specific gene content dynamics. Primarily, genes essential for virulence (i.e., type 1 secretion system [T1SS], T2SS and its effectors, T3SS and its effectors, T4SS, T6SS, adhesins, and *rpf* gene cluster) and adaptation (i.e., *gum*, iron uptake and utilization, xanthomonadin, and two-component systems) were depicted by comparative genomics of a *Xanthomonas* community comprising diverse lifestyles. Overall, the present analysis confers that nonpathogenic isolates of diverse hosts phylogenomically converge and are evolving in parallel with their pathogenic counterparts. Hence, there is a need to understand the world of nonpathogenic isolates from diverse and economically important hosts. Genomic knowledge and resources of nonpathogenic strains will be invaluable in both basic and applied research of the genus *Xanthomonas*.

**IMPORTANCE** Xanthomonas citri is one of the top phytopathogenic bacteria and is the causal agent of citrus canker. Interestingly, *Xanthomonas* is also reported to be associated with healthy citrus plants. The advent of the genomic era enabled us to carry out a detailed evolutionary study of a *Xanthomonas* community associated with citrus and other plants. Our genome-based investigations have revealed hidden and extreme interstrain diversity of nonpathogenic *Xanthomonas* strains from citrus plants, warranting further large-scale studies. This indicates an unexplored world of *Xanthomonas* from healthy citrus plant species that may be coevolving as a species complex with the host, unlike the variant pathogenic species. The knowledge and genomic resources will be valuable in evolutionary studies exploring its hidden potential and management of pathogenic species.

## Abstract (precis)

Xanthomonas citri is one of the top phytopathogenic bacteria and is the causal agent of citrus canker. Interestingly, *Xanthomonas* is also reported to be associated with healthy citrus plants. The advent of the genomic era enabled us to carry out a detailed evolutionary study of a *Xanthomonas* community associated with citrus and other plants. Our genome-based investigations have revealed hidden and extreme interstrain diversity of nonpathogenic *Xanthomonas* strains from citrus plants, warranting further large-scale studies. This indicates an unexplored world of *Xanthomonas* from healthy citrus plant species that may be coevolving as a species complex with the host, unlike the variant pathogenic species. The knowledge and genomic resources will be valuable in evolutionary studies exploring its hidden potential and management of pathogenic species.

## INTRODUCTION

*Xanthomonas* is a complex phytopathogen with an array of host ranges, including 268 dicots and 124 monocots (1–3). The genus comprises 34 different species with diversified pathogenic potential. *Citrus* spp. are widely known to be infected with Xanthomonas citri, a quarantine pathogen (4–7). Owing to its economic importance as one of the top phytopathogenic bacteria, its evolution into successful species is of interest to researchers, policy makers, etc. Moreover, it is the most successful *Xanthomonas* species, harboring 22 pathovars known to be host specific to diverse plants (8).

Interestingly, of these 34 species, only two species, i.e., Xanthomonas sontii and Xanthomonas maliensis, are exclusively nonpathogenic *Xanthomonas* strains associated with rice (NPXr) (9–12). Xanthomonas arboricola is known to comprise pathogenic strains of different pathovars affecting stone and nut fruit trees (13); nonpathogenic X. arboricola strains are also isolated from walnut trees (11, 12). Another well-studied nonpathogenic strain, Xanthomonas sacchari R1, was isolated from rice seeds and is known to be antagonistic to rice pathogens, which was later identified as a misclassified strain of *X. sontii* (14, 15). In addition to these, there are various unclassified and unexplored nonpathogenic isolates reported from a diversity of host plants (16). These host plants are already associated with successful pathogenic species of *Xanthomonas* and reported to cause devastating diseases worldwide. Although nonpathogenic strains have a significant impact on the evolution of *Xanthomonas* genus as a whole, they are widely overlooked due to their lower economic importance.

The genomic era has radically transformed our basic understanding of bacterial evolution. Phylogenomics and taxonogenomics are revealing the evolutionary processes that lead to genome diversification and speciation of the order *Lysobacterales*, comprising the genus *Xanthomonas* (17). *Xanthomonas* community analysis by comparative genomics of diverse lifestyles of bacteria is crucial for our understanding. The lifestyle and adaptability of a microbe to a new host or environment depends on its genomic content. In the genus *Xanthomonas*, there are widely known virulence-related and adaptation-related genes or gene clusters, such as type secretion systems and their effectors (15, 18–21), the *rpf* (regulation of pathogenicity factors) gene cluster (22, 23), iron uptake-, utilization-, and siderophore-related genes (24–26), global regulators and two-component systems (27–31), etc. The status of these genes or gene clusters among nonpathogenic strains of *Xanthomonas* will be of great significance. A genome-wide comparison of pathogenic and nonpathogenic isolates of X. arboricola reflects various genomic determinants, such as type 3 secretion systems (T3SS), chemoreceptors, adhesins, etc. (11). In this context, such an analysis of rice *Xanthomonas* community from our group has demonstrated the gene(s) or gene cluster(s) essential for virulence and adaptation to its host (15). Similarly, such a study on nonpathogenic *Xanthomonas* from diverse hosts of economically important citrus plants is lacking and is urgently needed.

In the present study, we have focused on the evolution of a citrus-associated *Xanthomonas* community known as a global devastating pathogen. Along with that, we also included genomes of nonpathogenic isolates from walnut and rice available from NCBI. Here, we sequenced four citrus-associated nonpathogenic *Xanthomonas* species complex (NPXc) strains and carried out phylogenomics, taxonogenomics, and comparative genomics studies. Primarily, we tried to address several important aspects, such as whether all these nonpathogenic counterparts from diverse hosts belong to a single species, irrespective of their host of isolation, or whether they belong to diverse species. What is their relation with two of the already known nonpathogenic species among those in the *Xanthomonas* genus? What is genomic content and flux of a gene(s)/gene cluster(s) during the course of evolution. The present study will be a genomic investigation towards understanding of widely unexplored nonpathogenic isolates and the evolution of successfully established pathogenic *Xanthomonas* species.

## RESULTS

### Whole-genome sequencing and assembly of NPXc isolates.

The genomes of four NPXc isolates were sequenced using an Illumina MiSeq platform. The assemblies obtained had coverage ranging from 73X to 146X with *N*_50_ values of 94 kb to 213 kb. Genome assembly assessment according to CheckM revealed all genomes to be approximately 99.9% complete with not more than 1.5% contamination. Genome size and GC content of all the strains are approximately 5 Mb and 68%, respectively. Annotation by NCBI resulted in 3,744 to 4,366 coding sequences with single copies each of 5S, 16S, and 23S in the rRNA operon. Genome statistics for the NPXc isolates are summarized in Table 1.

**TABLE 1:** Genome assembly statistics and metadata of NPXc strains in the present study

Strain	Host	Geographical location	Yr	Genome size (Mb)	Coverage (×)	No. of contigs	GC content (%)	*N*_50_ (kb)	No. of coding sequences	rRNA (5S, 16S, 23S)	tRNA	Completeness (%)/contamination (%)	NCBI accession no.
*Xanthomonas* sp. LMG8989	*Citrus* sp. (orange)	USA	1989	4.8	125	72	69.26	213	3,744	1,1,1	36	100/0.34	QUXI00000000
*Xanthomonas* sp. LMG8992	*Citrus* sp. (orange)	USA	1989	4.5	146	253	68.53	163	4,366	1,1,1	51	99.9/1.5	QUXJ00000000
*Xanthomonas* sp. LMG8993	*Citrus* sp. (orange)	USA	1989	4.9	93	78	65.82	139	4,141	1,1,1	51	99.9/0.5	QVIF00000000
*Xanthomonas* sp. LMG9002	*Citrus* sp. (orange)	USA	1989	4.9	73	94	68.80	94	3,987	1,1,1	51	100/0.34	QUXK00000000

### Phylogenomic and taxonogenomic diversity of nonpathogenic isolates.

**FIG 1:**
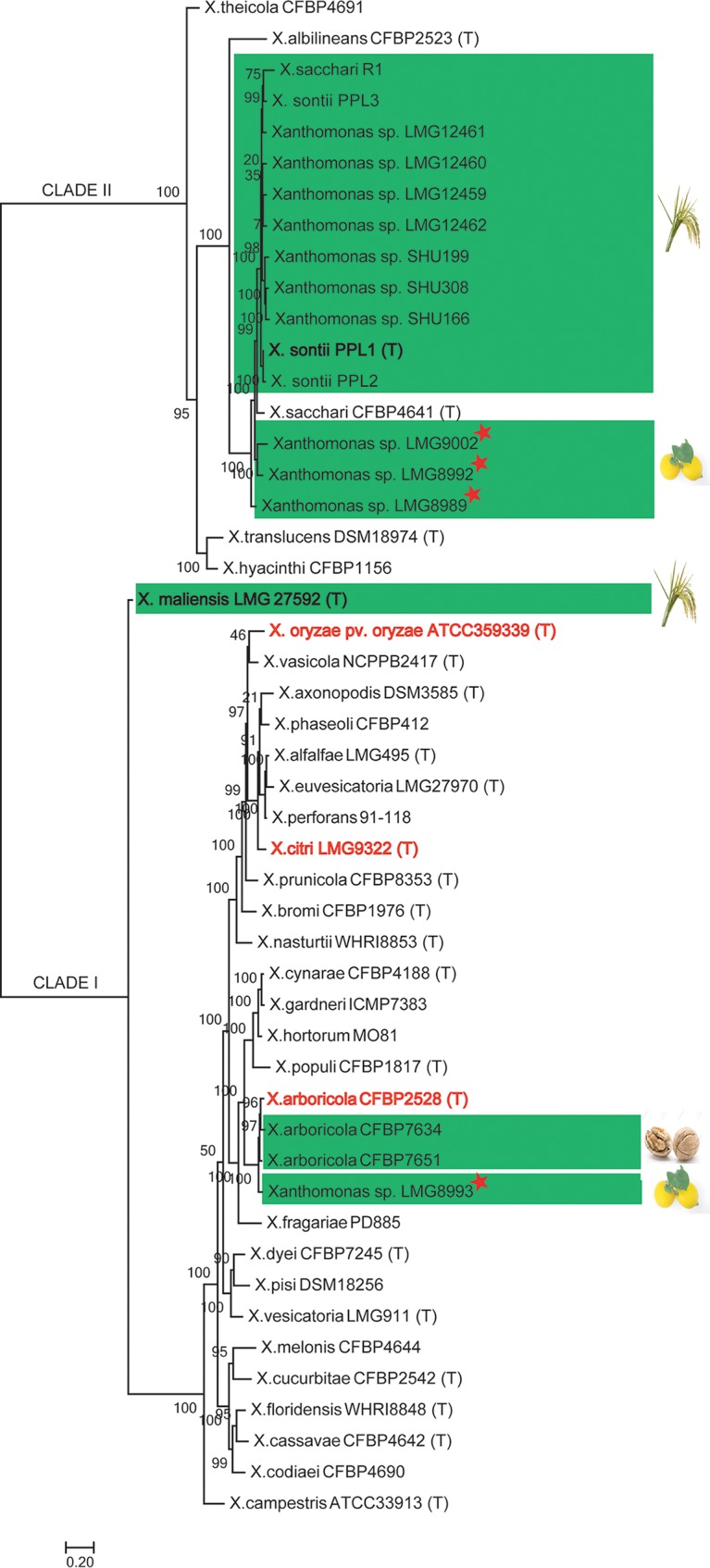
Phylogenomic tree of nonpathogenic and pathogenic *Xanthomonas* strains. Here, all the nonpathogenic strains are highlighted in green, and their respective hosts are shown. Pathogenic species *X. citri*, *X. oryzae*, and X. arboricola are highlighted in red. The type strains are designated by (T), and strains sequenced in the study are indicated by stars.

Phylogenomic analysis of all available nonpathogenic isolates (see Table S1 in the supplemental material) along with their pathogenic counterparts and other type or representative strains is shown in Fig. 1. The majority of the nonpathogenic strains, such as NPXc (LMG8989, LMG8992, and LMG9002) and NPXr (*X. sontii* PPL1, *X. sontii* PPL2, *X. sontii* PPL3, LMG12459, LMG12460, LMG12461, LMG12462, SHU166, SHU199, and SHU308) belong to clade II of the *Xanthomonas* genus. Interestingly, *X. sacchari* R1 was also with the NPXr strains in *X. sontii* and not in *X. sacchari*, whereas nonpathogenic isolates from walnut (NPXw; X. arboricola CFBP7634 and X. arboricola CFBP7651) and one of the NPXc (LMG8993) isolates belong to clade I, with X. arboricola as their closest phylogenomic relative. *X. maliensis* (NPXr) is more related to clade I but does not belong to any of the clades (Fig. 1).

10.1128/mSphere.00087-20.1 TABLE S1 Metadata of nonpathogenic strains used in the study. Download Table S1, XLSX file, 0.1 MB. Copyright © 2020 Bansal et al. 2020 Bansal et al. This content is distributed under the terms of the Creative Commons Attribution 4.0 International license.

To confirm the species status of these isolates, taxonogenomic analysis, including average nucleotide identity (OrthoANI) and digital DNA-DNA hybridization (dDDH), was performed. Here, NPXc, i.e., LMG8989, LMG8992, and LMG9002, had approximately 93% and 50% OrthoANI and dDDH, respectively, with *X. sontii* strains, and approximately 94% and 55%, respectively, with *X. sacchari* CFBP4641 (T). This suggests that none of the NPXc isolates belong to *X. sontii* or *X. sacchari*. Moreover, these three strains of NPXc had OrthoANI ranging from 92.7% to 93.7% and dDDH ranging from 48.9% to 52.7%. Therefore, these three strains represent three different potential novel species, which we refer to as the NPXc complex. The NPXc complex is phylogenomically related to *X. sontii*, which was reported earlier as a nonpathogenic species from rice (9). Furthermore, *X. sacchari* R1 has 96.6% OrthoANI and 69.7% dDDH with *X. sontii*, whereas these values are 94% and 54% with *X. sacchari*. This clearly depicts that *X. sacchari* R1 is a misclassified strain and that it belongs to *X. sontii*.

**FIG 2:**
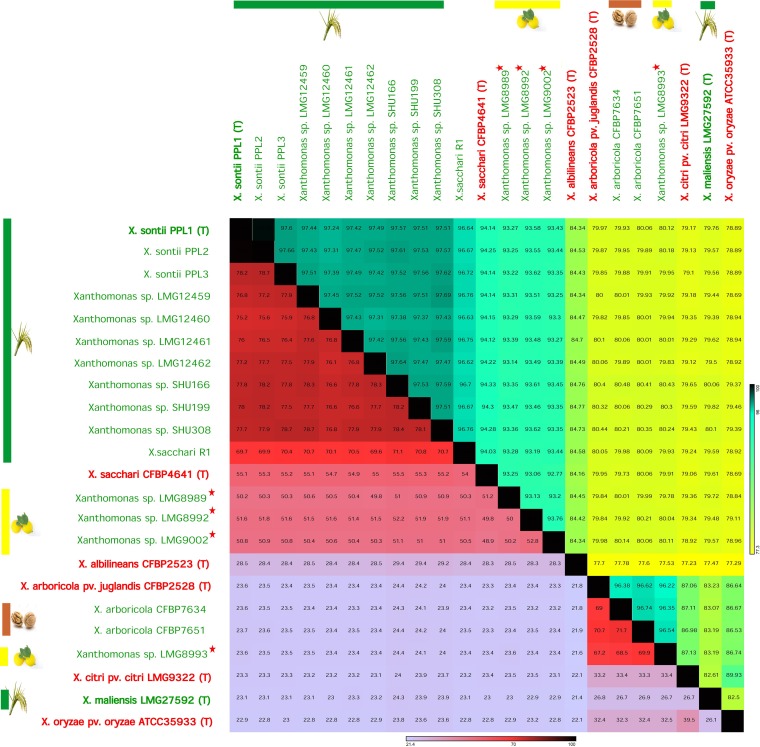
Taxonogenomic analysis of all nonpathogenic strains. Nonpathogenic and pathogenic strains are in red and green color, respectively. Nonpathogenic strains from rice, lemon, and walnut are represented by green, yellow, and brown bars, respectively. The type strains are designated by (T), and strains sequenced in the study are indicated by stars. Here, OrthoANI and dDDH values are shown in the upper and lower diagonal areas (separated by a line of black boxes), respectively.

Interestingly, the fourth NPXc isolate, LMG8993, and walnut-associated NPXw strains (CFBP7634 and CFBP7651) had around 96% OrthoANI but 67% dDDH among themselves. Hence, according to multiple genome similarity parameters, these three nonpathogenic isolates form a potential taxonomic outlier of pathogenic X. arboricola (Fig. 2). While NPXc and NPXr isolates are in both clades I and II, the NPXw isolates are only in clade I.

### *Xanthomonas*-associated gene cluster(s)/repertoire among bacteria with different lifestyles.

**FIG 3:**
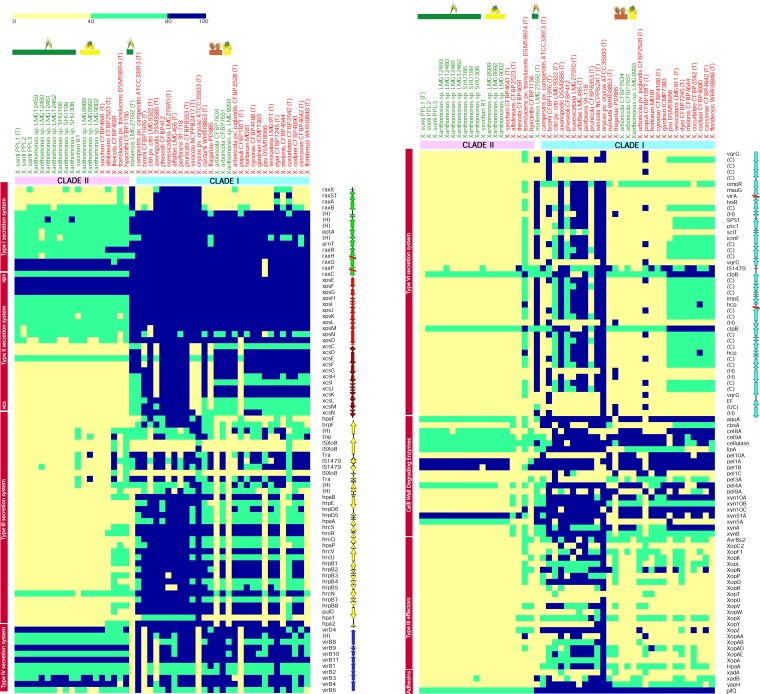
Heat maps showing status of pathogenicity-associated gene clusters (names indicated in vertical boxes, and vertical arrows indicate gene organization in the cluster). Here, contig breaks are represented by red lines for nonpathogenic strains (represented by green color) in the study and other species of the genus *Xanthomonas* (represented by red color). The intensity of the color indicates the level of identity with the query as depicted by the scale. (H), hypothetical gene; (C), conserved gene; (UC), uncharacterized gene; (T), transposase.

**FIG 4:**
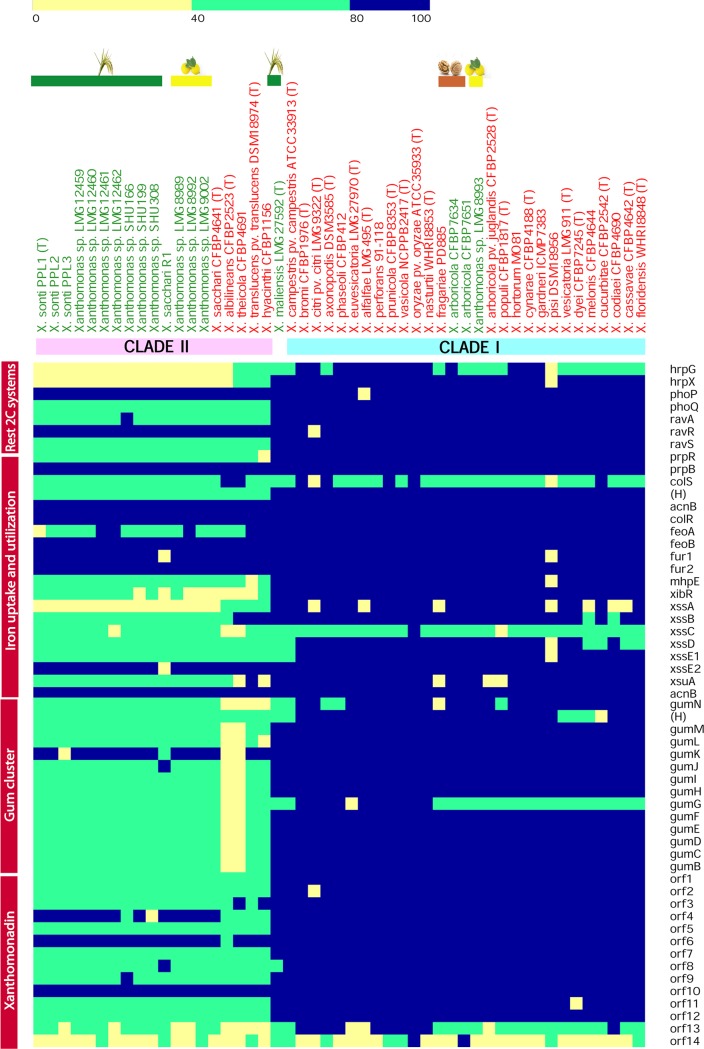
Heat map showing status of plant adaptation-related clusters (names indicated in vertical boxes) for nonpathogenic strains (represented by green color) in the study and other species of the genus *Xanthomonas* (represented by red color). The intensity of the color indicates the level of identity with the query as depicted by the scale. (H), hypothetical gene.

We further analyzed the status of a well-characterized *Xanthomonas*-associated gene cluster(s)/repertoire in these nonpathogenic isolates/species and their counterparts in the genus *Xanthomonas* (Fig. 3 and 4). Among the type secretion systems, T1SS, *xps* T2SS, T4SS, and *rpf* gene cluster were present in all the strains belonging to both clades (with exception of T4SS, which was absent from X. populi, X. oryzae, X. bromi, X. axonopodis of clade I, and X. maliensis). However, the remaining secretion systems were more prevalent in clade I than in clade II. For instance, *xcs* T2SS and T6SS were present in *X. maliensis* and most of the strains of clade I and absent from clade II (except for T6SS in X. translucens). Similarly, T3SS and its effectors were widely present in all strains of clade I except for X. floridensis, X. melonis, X. pisi, and nonpathogenic strains LMG8993 (NPXc) and CFBP7634 (NPXw). In case of the type III effectors, a diverse repertoire was observed for the strains isolated from diversified hosts. However, in clade II, T3SS and its effectors were present only in X. theicola, X. translucens, and X. hyacinthi. Furthermore, adhesins were also analyzed for the strains under study. Here, only *pilQ* was present in all the strains under study, and the remaining three adhesins, i.e., *xadA*, *xadB*, and *yapH*, were present in clade I, but their presence varied from species to species. Cell wall-degrading enzymes were present in all the isolates but with a diversified repertoire.

Overall, the pathogenicity cluster status among nonpathogenic isolates, T1SS, *xps* T2SS, T4SS, and the *rpf* cluster were present in nonpathogenic isolates of both clades. T3SS and its effectors and T6SS were absent from all the nonpathogenic strains except for one of the NPXw (CFBP7651) isolates having T3SS. T2SS *xcs* was present in nonpathogenic isolates from clade I only. Cell wall-degrading enzymes were present in both clade I and clade II nonpathogenic isolates. Here, clade II nonpathogenic isolates had similar repertoires, whereas clade I nonpathogenic isolates had diversified repertoires. Similarly, among the adhesin genes, clade I isolates were rich compared to clade II isolates.

Apart from studying if the status of gene clusters was virulence related, we further looked at the status of genes related to adaptation (Fig. 4). Accordingly, iron uptake and utilization, *gum* cluster, xanthomonadin pigment production cluster, and two-component system genes were present in all of the strains analyzed with few exceptions. For instance, *hrpG* and *hrpX* were absent from *X. pisi* and most of the strains of clade II (except for *X. theicola*, *X. translucens*, and *X. hyacinthi*). In the case of the *gum* cluster, it was missing from X. albilineans and *X. theicola* but present in all the strains of both clades and lifestyles.

### Gene content among nonpathogenic and pathogenic species of *Xanthomonas*.

Genome-wide studies of these ecological and phylogenomic relatives with diverse lifestyles enabled us to understand the evolution of highly successful lineages. To evaluate gene content differences among nonpathogenic strains (NPXc, NPXr, and NPXw) and their pathogenic counterparts, i.e., those isolated from the host (*X. citri*, *X. oryzae*, and X. arboricola), we performed pan genome analysis. Here, 1,322 and 12,094 genes constituted core and pan genome, respectively, for all the strains from distinct lifestyles. However, only 1 and 12 genes were found to be present in all nonpathogenic and pathogenic strains, respectively. This low number of core genes can be explained by the fact that even though strains belong to same lifestyle, phylogenomically, they are diverse, i.e., they belong to distinct clades and hosts.

**FIG 5:**
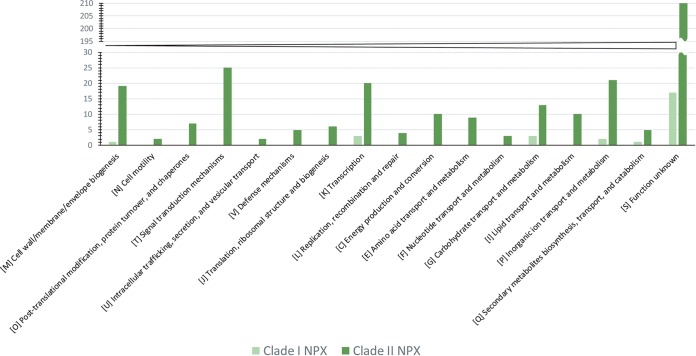
Distribution of COG-based functional categories of nonpathogenic isolates of clade I and clade II.

Hence, to get lifestyle-specific genes, we analyzed gene content common to all the nonpathogenic strains belonging to a particular clade. Interestingly, for the clade II strains, i.e., LMG8989, LMG8992, LMG9002, *X. sontii* PPL1, *X. sontii* PPL2, *X. sontii* PPL3, LMG12459, LMG12460, LMG12461, LMG12462, SHU166, SHU199, SHU308, and *X. sacchari* R1, there were 377 core genes (see Table S2). Similarly, there were 27 core genes for nonpathogenic strains from clade I, i.e., LMG8993, X. arboricola CFBP7634 and X. arboricola CFBP7651 (see Table S3). This clearly shows nonpathogenic strains of clade II are diversifying rapidly compared to clade I nonpathogenic strains. All these genes were further subjected to Clusters of Orthologous Groups (COG) functional classification. Of 25 COG classes, clade II nonpathogenic core genes were classified in 17 COG classes, and clade I nonpathogenic core genes were only in 6 COG classes (Fig. 5). Among clade II nonpathogenic isolates, genes were classified under “signal transduction mechanisms,” “transcription,” “cell wall/membrane/envelope biogenesis,” etc., whereas clade I nonpathogenic isolates belonged to “transcription,” “carbohydrate and inorganic ion transport and metabolism,” and “secondary metabolites biosynthesis, transport, and catabolism.”

10.1128/mSphere.00087-20.2 TABLE S2 Genes unique to nonpathogenic strains of clade II. Download Table S2, XLSX file, 0.1 MB. Copyright © 2020 Bansal et al. 2020 Bansal et al. This content is distributed under the terms of the Creative Commons Attribution 4.0 International license.

10.1128/mSphere.00087-20.3 TABLE S3 Genes unique to nonpathogenic strains of clade I. Download Table S3, XLSX file, 0.1 MB. Copyright © 2020 Bansal et al. 2020 Bansal et al. This content is distributed under the terms of the Creative Commons Attribution 4.0 International license.

**FIG 6:**
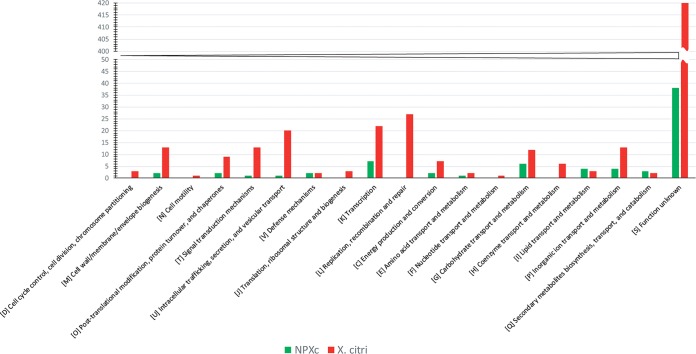
Distribution of COG-based functional categories of citrus-associated nonpathogenic and pathogenic isolates.

To look for lifestyle-specific genes among citrus associated strains of *Xanthomonas*, we carried out pan genome analysis, including 4 NPXc (LMG8989, LMG8992, LMG8993, and LMG9002) and 4 *X. citri* pv. citri strains (LMG9322, XACB100, XACJJ10, and XALG98) (8, 32). Here, 2,344 and 7,736 genes constituted core genome and pan genome, respectively. Furthermore, 585 and 78 genes, respectively, were present in all pathogenic and nonpathogenic strains (see Tables S4 and S5). These genes were also then COG classified, and their distribution is shown in Fig. 6. Functional classification suggested that pathogenic isolates had a rich unique gene repertoire compared to that of nonpathogenic isolates. Pathogenic strains had unique genes related to “replication, recombination, and repair,” “transcription,” “intracellular trafficking, secretion, and vesicular transport,” and “cell wall/membrane/envelope biogenesis.” However, the genes unique to NPXc isolates belonged to “transcription” and “carbohydrate, lipid, and inorganic ion transport and metabolism.”

10.1128/mSphere.00087-20.4 TABLE S4 Genes unique to pathogenic strains *Citrus* sp. (*X. citri* pv. citri). Download Table S4, XLSX file, 0.1 MB. Copyright © 2020 Bansal et al. 2020 Bansal et al. This content is distributed under the terms of the Creative Commons Attribution 4.0 International license.

10.1128/mSphere.00087-20.5 TABLE S5 Genes unique to *Citrus*-associated nonpathogenic strains. Download Table S5, XLSX file, 0.1 MB. Copyright © 2020 Bansal et al. 2020 Bansal et al. This content is distributed under the terms of the Creative Commons Attribution 4.0 International license.

## DISCUSSION

*Xanthomonas* pathogens of citrus and rice belong to *X. citri* and *X. oryzae*, respectively (7, 8, 33). In this context, it is interesting to find that nonpathogenic isolates from citrus are highly diverse, with each isolate belonging to a potentially novel species and altogether forming a species complex, the NPXc complex. This suggests the existence of a highly diverse and coevolving community of nonpathogenic *Xanthomonas* on citrus plants. This is unlike nonpathogenic isolates from rice that primarily belong to one species, *X. sontii*, suggesting distinct selection pressure and diversification in nonpathogenic isolates depending on their hosts. Owing to the diversity of NPXc, there is a need to expand such studies by isolating and investigating large numbers of nonpathogenic strains from citrus plants. Incidentally, unlike rice, citrus plants themselves belong to several highly diverse species. Hence, such studies are required to be expanded to all citrus species and not just one species to understand the origin, evolution, and adaptation of a nonpathogenic population on citrus hosts.

Most of the nonpathogenic isolates in both rice and citrus belong to clade II. At the same time, a minority of the strains in both rice (*X. maliensis*), citrus (LMG8993), and even *Xanthomonas*-associated with walnut (NPXw; CFBP7634 and CFBP7651) were found to be more related to pathogenic isolates. This suggests the existence of a minor group that may be ancestral to the pathogenic counterparts and ongoing dynamics toward diverse lifestyles. Also, pathogenic *X. citri* and X. arboricola species are known to cause similar types of symptoms in diverse hosts (34). One of the nonpathogenic isolates from citrus (LMG8993) is related to pathogenic and nonpathogenic isolates of walnut, indicating the basic functions required to adapt to fruit plants. Moreover, a previous study comparing pathogenic and nonpathogenic strains from walnut found that they belong to the same species (X. arboricola). Interestingly, whole-genome-based comparisons have shown that NPXw isolate (CFBP7651, also included in the present study) had noncanonical T3SS (11). Similarly, among NPXr isolates as well, *X. maliensis* was found to be more related to pathogenic isolates and also had some of the key pathogenicity determinants which were otherwise absent from the majority of nonpathogenic isolates (15).

Phylogenetically pathogenic species of rice (*X. oryzae*) and citrus (*X. citri* pv. citri) are distantly related (35), and unlike nonpathogenic isolates, both of the pathogenic species fall in a single clade (clade I). This may be to do with the pathogenic lifestyle on respective hosts and the requirement of distinct set of virulence genes. Hence, a nonpathogenic lifestyle may have been the default or original lifestyle. During the course of evolution and with the advent of agriculture or anthropological factors, this might have led to the emergence of variant pathogenic species or strains. Such emergence might have been from a nonpathogenic *Xanthomonas* strain which is not successful, such as that seen in *X. maliensis* that is also intermediate to both nonpathogenic and pathogenic strains in relationship and gene content. Even though the nonpathogenic strains are from two diverse hosts (monocot versus dicot), in the phylogenomic tree, the majority of the isolates were closely related owing to their nonpathogenic lifestyle. This highlights that the origin of both communities is from a common ancestor in the recent past and that lifestyle is also important along with host specialization. There is parallel evolution and convergence of function related to this lifestyle. This also means there is requirement for a core set of functions to be a successful nonpathogenic *Xanthomonas*.

Earlier molecular genetic studies have identified many genes/gene clusters that are required for pathogenicity in *Xanthomonas*. Our genomic investigation studies also allowed us to demarcate genes/gene clusters that might have played important roles in the evolution of a pathogenic lifestyle, such as T3SS, *rpf*-based gene regulation, *gum* cluster for exopolysaccharide production, xanthomonadin pigment production, etc., and are core to both lifestyles of *Xanthomonas*. These findings suggest that the functions required for plant adaptation, primarily in nonpathogenic strains, is at the level of the epiphyte for their establishment. The pathogenic lifestyle is more aggressive and requires functions at all stages until plant death. This observation is also supported by pan genome analysis that indicates the need to acquire a large number of unique genes with unknown and diverse functions for a successful pathogen. The dominance of genes related to replication, repair, and recombination reiterates the importance of horizontal gene transfer in the acquisition of a large number of genes in *Xanthomonas* strains that are pathogenic to citrus. The dominance of transcription category genes also suggests that apart from diversification and acquisition of novel genes through horizontal gene transfer, regulation and on-going variation in the core genes is also important.

Hence, the present study reflects that phylogenomically and gene content wise, nonpathogenic strains have two waves of evolution. The first one is followed by a majority of the strains, forming a species complex with the absence of virulence-related clusters but basic adaptation functions. The second one is more diversified and related to pathogenic strains, although they are represented by a minority of the strains. Resource and knowledge pertaining to the population of nonpathogenic *Xanthomonas* from diverse hosts will shed light on the epidemiology and evolution of pathogenic isolates. Genomics has lead to a new era in *Xanthomonas* research by enabling researchers to carry out large-scale comprehensive phylogenomic profiling to understand evolutionary differences in both minor and major groups of nonpathogenic isolates across diverse hosts, such as grasses, shrubs, trees, etc.

## MATERIALS AND METHODS

### Strain collection, genome sequencing, assembly and annotation.

All four citrus-associated strains were procured from BCCM-LMG culture collection and were grown as per the instructions. Genomic DNA was extracted by using a ZR Fungal/Bacterial DNA MiniPrep kit (Zymo Research). DNA quality checking was carried out using a NanoDrop 1000 instrument (Thermo Fisher Scientific) and agarose gel electrophoresis. Quantitation of DNA was performed using a Qubit 2.0 fluorometer (Life Technologies). Illumina paired-end sequencing libraries (read length, 2 × 250 bp) of genomic DNA were prepared using Nextera XT sample preparation kits (Illumina, Inc., San Diego, CA, USA) with dual indexing adapters. Illumina sequencing libraries were sequenced in-house using an Illumina MiSeq platform (Illumina, Inc., San Diego, CA, USA) and company-supplied paired-end sequencing kits. Adapter trimming was performed automatically by MiSeq control software (MCS), and additional adapter contamination identified by the NCBI server was removed by manual trimming. Raw reads were assembled *de novo* using CLC Genomics Workbench v7.5 (CLC bio, Aarhus, Denmark) with default settings. The quality of the genomes was accessed using CheckM v1.0.13 (36). Annotation was performed using the NCBI Prokaryotic Genome Annotation Pipeline (PGAP).

### Phylogenomics and taxonogenomics.

PhyloPhlAn v0.99 was used to construct the phylogenomic tree based on more than 400 conserved genes from whole-genome proteome data (37). USEARCH v5.2.32 (38), MUSCLE v3.8.31 (39), and FastTree v2.1 (40) were used for ortholog searching, multiple-sequence alignment, and phylogenomic construction, respectively. Taxonogenomic analysis of the strains was performed using the OrthoANIu (41) standalone tool, which employs USEARCH v5.2.32 over BLAST for OrthoANI calculation, and dDDH values were calculated using a genome-to-genome distance calculator (http://ggdc.dsmz.de/distcalc2.php).

### Pan genome analysis.

Pan genome analysis of the strains was performed using Roary v3.12.0 (42). Here, gff files generated by Prokka v1.13.3 (43) were used as input for Roary. Since we were analyzing genomes from different species, the identity cutoff used was 60%. Functional annotation of the core genes identified was performed using EggNOG mapper v1 (44).

### Virulence related gene(s) and gene cluster(s) analysis.

Protein sequences of genes or gene clusters (15) were used as the query, and tBLASTn searches were performed on genomes. Genomic coordinates corresponding to the gene clusters were identified from tBLASTn, and corresponding gene clusters from the genomes were extracted using SAMtools v1.9 faidx module (45). Here, tBLASTn searches were performed using standalone BLAST+ v2.9.0 (46), and cutoffs used for identity and coverage were 40% and 50%, respectively. Heat maps for the blast searches were generated using GENE-E v3.03215 (https://software.broadinstitute.org/GENE-E/).
